# Supplementary material for: Contrasting pathways to tree longevity in gymnosperms and angiosperms
Source: Nat Commun. 2025 Dec 19;17:898. doi: 10.1038/s41467-025-67619-2 (PMC12830884; doi:10.1038/s41467-025-67619-2)
Supplement: Supplementary file 1 — Supplementary Information [file 41467_2025_67619_MOESM1_ESM.pdf]

Supplementary info for:

Contrasting pathways to tree longevity in gymnosperms and angiosperms

Authors

Roel J. W. Brienen[1*], Giuliano Maselli Locosselli[2], Stefan Krottenthaler[3], Emanuel Gloor[1], Robyn Wrigley[4], Steven L. Voelker[5], Jan Altman[6,7], Nela Altmanova[6,8], Leander D. L. Anderegg[9], Michele Baliva[10], Deepak Barua[11], Vaclav Bazant[7], Bryan Black[12], Peter M. Brown[13], Gregorio Ceccantini[14], R. Justin DeRose[15], Jose Villanueva Diaz[16], Alfredo Di Filippo[17], Jiri Dolezal[6,8], Louis Duchesne[18], Christopher Earle[19], Pavel Fibich[6,8], Hardy Griesbauer[20], Samuli Helama[21], Stefan Klesse[22], Kirill Korznikov[6], David Lindenmayer[23], Shuhui Liu[24], Lidio Lopez[25], Maurizio Mencuccini[26,27], Thomas A. Nagel[28], Jakob Pavlin[7], Neil Pederson[29, 30], Gianluca Piovesan[10], Christina Restaino[31], Peter B. Reich[32, 33], David Sauchyn[34], Jochen Schöngart[35], John D Shaw[36], Dan Smith[37], Ron Sunny[38], Miroslav Svoboda[7], Ricardo Villalba[25], Lisa J. Wood[39], Chunyu Zhang[24]

Affiliations

1 University of Leeds, School of Geography, Leeds, LS2 9JT, UK
2 University of São Paulo, Center of Nuclear Energy in Agriculture, Piracicaba, Brazil
3 University of Passau, Physical Geography, Passau, Germany
4 University of Leeds, School of Earth and Environment, Leeds, LS2 9JT, UK
5 College of Forest Resources and Environmental Science, Michigan Technological University, Houghton, Michigan, USA
6 Institute of Botany of the Czech Academy of Sciences, Dukelská 135, 379 01 Třeboň, Czech Republic
7 Faculty of Forestry and Wood Sciences, Czech University of Life Sciences, Prague, Kamýcká 129, 165 21, Prague 6 - Suchdol, Czech Republic
8 Faculty of Science, University of South Bohemia, 370 05 České Budějovice, Czech Republic
9 University of California Santa Barbara, Department of Ecology, Evolution & Marine Biology. Santa Barbara, CA, USA 93106
10 Università della Tuscia, Department of ecological and biological science (DEB), Viterbo, Italy
11 Department of Biology, Indian Institute of Science Education and Research, Pune, India
12 Laboratory of Tree Ring Research, University of Arizona, Tucson, AZ, 85721, USA
13 Rocky Mountain Tree-Ring Research, 2901 Moore Lane Fort Collins Colorado 80526 USA
14 University of São Paulo, Institute of Biosciences, Dpt of Botany, Rua do Matão, 277, 05806-090, São Paulo, SP, Brazil
15 Department of Wildland Resources and Ecology Center, 5230 Old Main Hill, Logan, Utah, USA 84322-5230
16 Laboratorio de Dendrocronologia, Instituto Nacional de Investigaciones Forestales, Agricolas, y Pecuarias, Gomez Palacio, Mexico
17 Università della Tuscia, Department of Agriculture and Forest Science (DAFNE), Viterbo, Italy
18 Ministère des Ressources naturelles et des Forêts, Direction de la recherche forestière, 2700 Einstein Street, Quebec City, Quebec, G1P 3W8, Canada.
19 Gymnosperm Database, Olympia WA USA
20 British Columbia Ministry of Forests, 499 George St, Prince George, BC, V2L 1R5, Canada
21 Natural Resources Institute Finland, 96200 Rovaniemi, Ounasjoentie 6, Finland
22 Forest and Soil Ecology, Swiss Federal Institute for Forest, Snow and Landscape Research, WSL, 8903 Birmensdorf, Switzerland
23 Fenner School of Environment and Society, The Australian National University, Canberra, ACT, 2601, Australia
24 Research Center of Forest Management Engineering of State Forestry and Grassland Administration, Beijing Forestry University, Beijing, China
25 Laboratorio de Dendrocronología e Historia Ambiental, IANIGLA/CONICET-Mendoza, Argentina
26 CREAF, Bellaterra, Spain
27 ICREA, Barcelona, Spain.
28 Department of forestry and renewable forest resources, University of Ljubljana, Ljubljana, Slovenia
29 Independent Scholar, Maynard, MA, USA
30 Harvard Forest, Harvard University, Petersham, MA, USA
31 University of Nevada, Reno, Reno Nevada 89502
32 Institute for Global Change Biology, University of Michigan, 440 Church Street, Ann Arbor, MI 48109, USA
33 Department of Forest Resources, University of Minnesota, St. Paul, MN, USA
34 Prairie Adaptation Research Collaborative, Geography and Environmental Studies, University of Regina, Canada
35 Instituto Nacional de Pesquisas da Amazônia (INPA), Ecologia, Monitoramento e Uso Sustentável de Áreas Úmidas (MAUA), Av. André Araújo 1756, 69011-910 Manaus-AM, Brazil
36 Rocky Mountain Research Station, USDA Forest Service, Ogden, Utah, USA
37 Department of Geography, University of Victoria, PO Box 1700 STN CSC, Victoria, BC V8W 2Y2, Canada
38 Department of Botany, St Joseph's College (Autonomous), Devagiri, Calicut, Kerala, India
39 University of Northern British Columbia, Faculty of Environment, 3333 University Way, Prince George, BC, Canada, V2N 4Z9

***Corresponding author : Roel J. W. Brienen, r.brienen@leeds.ac.uk**

Supplementary Table 1. Bivariate relationships between log_{10}-transformed species' longevity and environmental variables and species' traits for the full dataset, and for angiosperm and gymnosperm species separately. Shown are the Pearson's correlation coefficients and uncorrected p-values (i.e., no Bonferroni correction for multiple comparisons).

		Full dataset			Angiosperms			Gymnosperms		
		Pearson R	p value	n	Pearson R	p value	n	Pearson R	p value	n
Climate	Mean annual temperature	-0.255	0.000	739	0.030	0.488	534	-0.378	0.000	205
	Growing season temperature	-0.283	0.000	716	0.015	0.742	516	-0.383	0.000	200
	Precipitation driest quarter**	0.153	0.000	691	0.332	0.000	488	0.080	0.258	203
	Mean annual precipitation**	0.029	0.439	691	0.251	0.000	488	-0.036	0.613	203
	Vapour pressure Deficit	-0.235	0.000	718	-0.004	0.920	517	-0.255	0.000	201
	Climate moisture index**	0.150	0.000	671	0.266	0.000	469	0.107	0.129	202
Environment	Growing season length	-0.139	0.000	716	0.153	0.001	516	-0.326	0.000	200
	Net Primary productivity	-0.167	0.000	718	0.115	0.009	517	-0.359	0.000	201
	Proximity to the tree line	0.279	0.000	720	-0.071	0.108	519	0.397	0.000	201
Soil	Soil Cation Exchange Capacity	0.151	0.000	716	-0.142	0.001	513	0.222	0.001	203
	Soil pH	-0.018	0.629	716	-0.256	0.000	513	0.046	0.516	203
Traits										
Growth	Mean radial growth*	-0.553	0.000	613	-0.564	0.000	429	-0.567	0.000	184
Plant stature	Maximum tree height	0.324	0.000	523	0.351	0.000	325	0.200	0.005	198
Wood	Wood density	-0.092	0.025	585	0.124	0.009	441	-0.081	0.333	144
Hydraulic	Conduit density	0.289	0.000	202	0.114	0.132	177	0.096	0.647	25
	Conduit diameter*	-0.494	0.000	165	-0.123	0.250	89	-0.197	0.088	76
	P50	-0.149	0.009	304	0.034	0.633	199	0.160	0.102	105
	Hydraulic safety margin (HSM)	0.234	0.002	167	0.016	0.862	115	0.211	0.133	52
	Stem hydraulic conductivity	-0.139	0.033	233	0.044	0.608	141	-0.026	0.805	92
Leaf	Leaf Mass per Area*	0.402	0.000	163	-0.048	0.599	124	0.175	0.287	39
	Nitrogen per Mass	-0.506	0.000	164	-0.284	0.001	124	0.009	0.957	40
	Photosynthetic assimilation per mass	-0.405	0.000	101	0.043	0.717	72	-0.196	0.308	29
	Leaf longevity	0.355	0.000	97	0.012	0.919	73	0.107	0.618	24
Reproduction	SeedMass	-0.104	0.209	149	-0.004	0.969	88	-0.016	0.900	61

* These data were log_{10}-transformed for the analysis; ** Correlations with precipitation and Climate moisture index exclude species that have their main occurrence in floodplains or wetlands.

Supplementary Table 2 Standardized coefficients of the relationships between species longevity and climate and soil characteristics. Statistics were performed using multiple linear regression analysis. Shown are the standardised effects, the p values, and the explained variances. from the R package relaimpo, v2.2.7, as recommended by ref. 1.

		Angiosperms (n=469)		Gymnosperms (n=197)	
		Beta (stand)	Explained variance	Beta (stand.)	Explained variance
Climate	Mean Annual Temperature	-0.13 (p=0.036)	0.6%		
	Growing season length			-027 (p<0.001)	8.5%
	Growing season temperature			-0.30 (p<0.001)	12.8%
	Precipitation driest quarter	0.32 (p<0.001)	10.3%	0.23 (p=0.013)	1.9%
Soil	Soil Cation Exchange Capacity	-0.22 (p<0.001)	2.7%		
	Full model		13.7%		23.8%

Supplementary Table 3 Overview of datasets

Supplementary Table 3a Key datasets

Data source	Locations	Number of sites	Number trees	Number species	Link
National Forestry Inventory - Quebec	Quebec, Canada	68.316	238.145	12	MRNF
International Tree-Ring Data Bank (ITRDB)	Global	4.780	199.397	242	https://www.ncei.noaa.gov/products/paleoclimatology/tree-ring
Zhang, unpublished	Northeastern China	2.317	21.295	51	
Remote Primary Forest, unpublished	South, central Europe	1.650	22.196	10	https://www.remoteforests.org/project.php?language=en
Altman laboratory, unpublished	Global	600	5.439	56	
DENDROAM, Schoengart unpublished	Amazon	92	1.750	54	
Other (contributors, literature)	Global	2.313	50.538	406	
Additional online data sources:					
Oldlist (Peter Brown)	Global				http://www.rmtrr.org/oldlist.htm
Eastern Oldlist (Neil Pederson)	Global				https://www.ldeo.columbia.edu/~adk/oldlisteast/
Eastern Native Tree Society (ENTS)	Global				http://www.nativetreesociety.org/dendro/ents_maximum_ages.htm
Ontario Old growth forest (Michael Henry)	Global				https://www.oldgrowth.ca/oldtrees/

Supplementary Table 3b Age estimation by method

Method	Number species	Percent
Tree ring record	576	78%
Growth projection	120	16%
Radiocarbon	37	5%
Historical information	6	1%

Supplementary Table 3c Longevity confidence levels

Confidence level	Number species (percent)	Methods	Minimum # sites, samples	Description
1 – Lowest confidence	332 (45%)	Tree rings	1 site, 10 trees/series[1]	Published reports of age estimates based on tree ring counts on 10 trees/series.
2 – Moderate confidence	163 (22%)	Growth projections<br><br>Radiocarbon dating<br>Historical records	Not applicable<br><br><br>1 large tree<br><br>1 large tree	Estimates based on maximum tree size and growth, or mortality rates<br><br>Single radiocarbon date<br><br>Historical record
3 – High	50 (7%)	Tree rings	1 site, 150 trees[2]	Includes expert verified age records from well-studied species in e.g. Oldlists
4 – Very high	194 (26%)	Tree rings	3 sites,150 trees[1]	Verified tree ring counts obtained from original data (ITRDB, NFI, collaborators, or peer-reviewed publications)

[1]Minimum sample size for determining estimates of tree lifespan close to true longevity.
[2]This category includes species with data from literature/collaborators with less than 100 samples in total, but where we know the species has been used widely in tree ring studies.

Supplementary Table 4. Functional traits and the database origin.

Functional trait	Unit	Description	Database(s)
Maximum tree height (H_{max})	m	Maximum tree height measured from trunk base to top of the canopy	Tallo[2] https://zenodo.org/record/6637599 ; The Gymnosperm Database, www.conifers.org; Monumentaltrees.com
Wood density (WD)	kg/m^3	Dry mass of a unit of volume of wood	Global wood density database[3]; CIRAD wood density[4], https://doi.org/10.18167/DVN1/KRVF0E DENDROAM (DENDROecological database of the Amazon, Schoengart, 2024, unpublished) Individual records from various sources
Conduit density (VD)	Number per mm^2	Density of vessels (angiosperm), or tracheids (gymnosperm)	Refs. 5, 6 and 7 https://doi.org/10.5061/dryad.1138
Conduit diameter	µm	Mean tangential vessel diameter (angiosperm), or tracheid diameter (gymnosperm)	
P50	MPa	The xylem water potential at which 50% of the maximum hydraulic conductance is lost.	Xylem Functional Traits database, preliminary version from ref. 6. Unpublished tropical data, Sunny and Barua, 2024
Hydraulic safety margin (HSM)	MPa	Difference between the minimum observed water potential in the xylem and P50	
Leaf mass per area (LMA)	g/m^2	Leaf dry mass per area	GLOPNet leaf economics dataset[8]
Nitrogen per leaf mass (N_{mass})	%	Leaf nitrogen per mass	
Photosynthetic assimilation per mass (A_{mass})	nmol $g^{-1}s^{-1}$	Photosynthetic assimilation rates measured under high light, ample soil and ambient CO_2	
Leaf longevity	Months	Leaf longevity	
Seedmass	mg	Mean seed dry mass	TRY plant trait database_ request No 30569[9]

a.

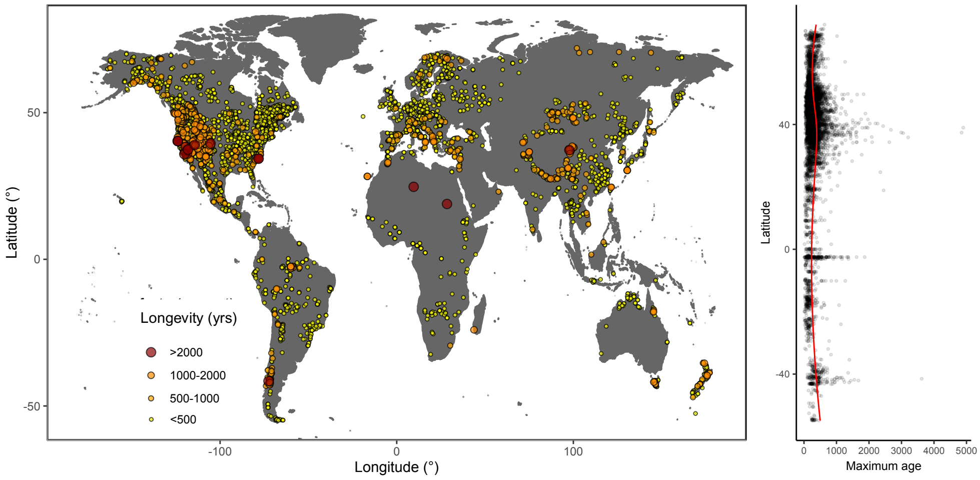

b.

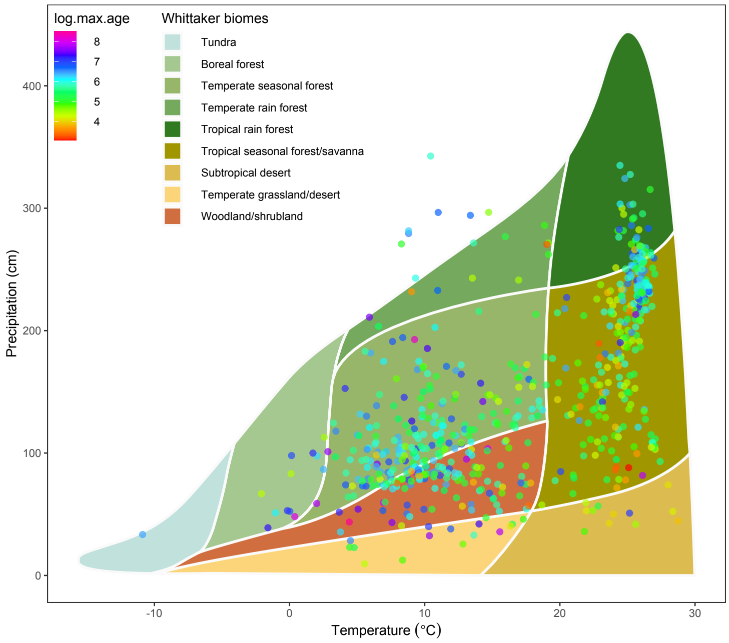

Supplementary Figure 1. Map of all study sites (a), and plot of the species included in this study onto Whittaker Biome plot (b). Note we excluded data from Quebec NFI from the map. The plot biome was produced using R package plotbiomes[10] (see https://doi.org/10.5281/zenodo.7145245).

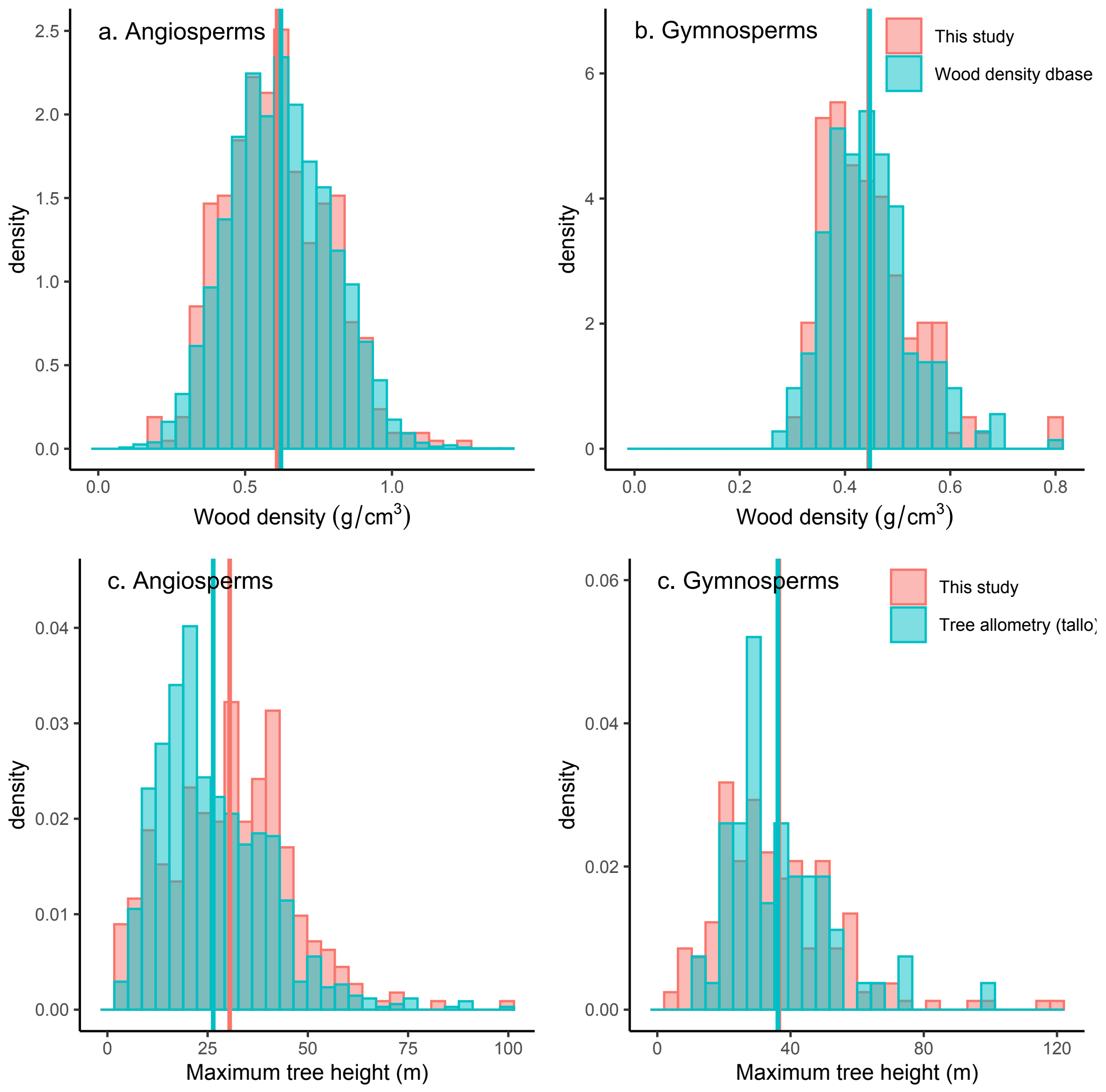

Supplementary Figure 2. Comparison of wood density (a,b) and maximum tree height (c,d) of the sampled species in this study to that of global functional trait datasets. For wood density we used the global wood density database[3] and for maximum tree height we used tallo, a tree allometry database[2]). The vertical lines indicate the means of the two datasets. The mean wood density (WD) of our study (angiosperms= 0.61 g/cm^3, gymnosperms = 0.45 g/cm^3) compares well to that from the global wood density database (angiosperms=0.62 g/cm^3, 8,150 species, gymnosperms = 0.45 g/cm^3, 262 species) and to the community weighted wood density from reference [11] (angiosperms=0.59 g/cm^3, 8,036 species; gymnosperms = 0.47 g/cm^3, 213 species). The maximum tree height of our study (angiosperms= 30.5 m, gymnosperms =36.2 m) compares well to that from the tallo database[2] when restricted to those species with 50 records or more (angiosperms=26.4 m, 992 species, gymnosperms = 36.5 m, 65 species).

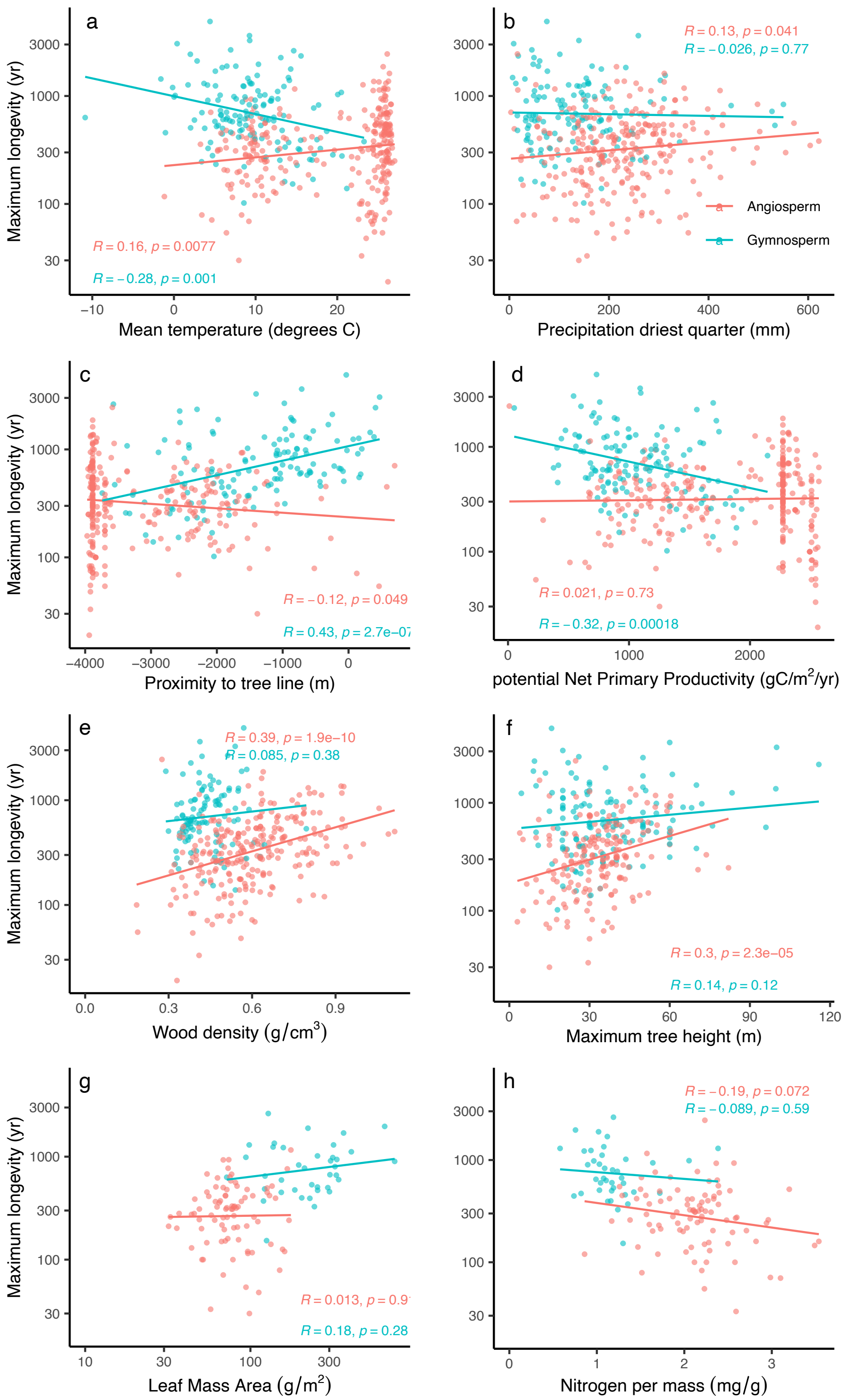

Supplementary Figure 3 Relationships between species' longevity and most important climate, environmental and trait variables including only confidence category 2 and higher. These graphs exclude tree ring estimates based on fewer than 150 species (see methods and ED Table 3c) Variables include species' mean annual temperature (a) and precipitation during the driest quarter (b), species' mean proximity to the estimated tree line position (c), potential net primary productivity (d), and species' functional traits including wood density (e), maximum species tree height (f), and leaf mass per area (g) and leaf nitrogen (h). Trend lines are fitted using a linear model. Statistics show the correlation coefficient (*R*) and significance levels (*p*) for the linear relationships. Data were grouped by the two major taxonomic groups in the dataset, angiosperms and gymnosperms. Note that data for Leaf Mass per Area were log_{10} transformed.

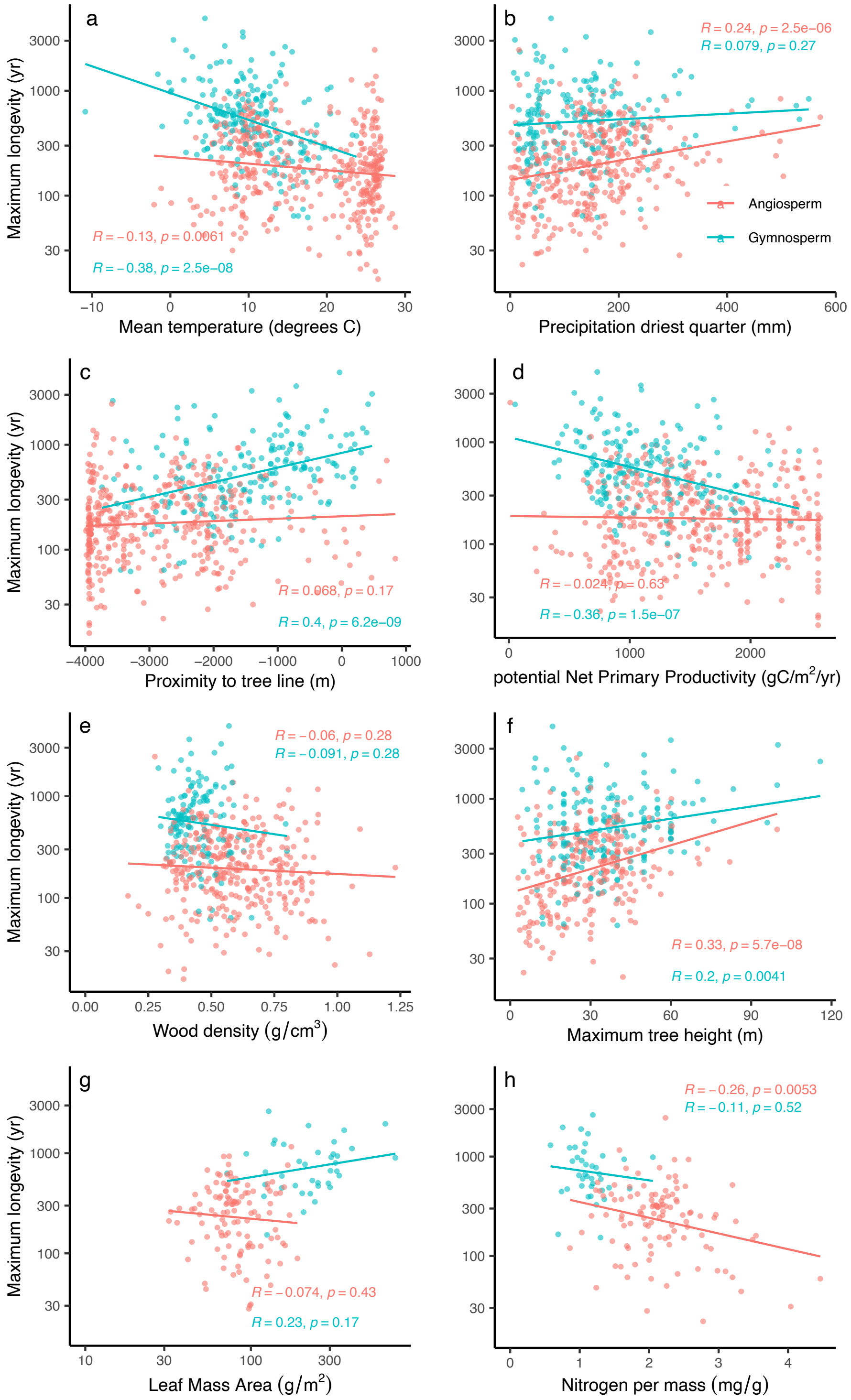

Supplementary Figure 4. Relationships between species' longevity and most important climate, environmental and trait variables excluding longevity data based on growth projections (see methods and ED Table 3c). Variables include species' mean annual temperature (a) and precipitation during the driest quarter (b), species' mean proximity to the estimated tree line position (c), potential net primary productivity (d), and species' functional traits including wood density (e), maximum species tree height (f), and leaf mass per area (g) and leaf nitrogen (h). Trend lines are fitted using a linear model. Statistics show the correlation coefficient (*R*) and significance levels (*p*) for the linear relationships. Data were grouped by the two major taxonomic groups in the dataset, angiosperms and gymnosperms. Note that data for Leaf Mass per Area were log_{10} transformed.

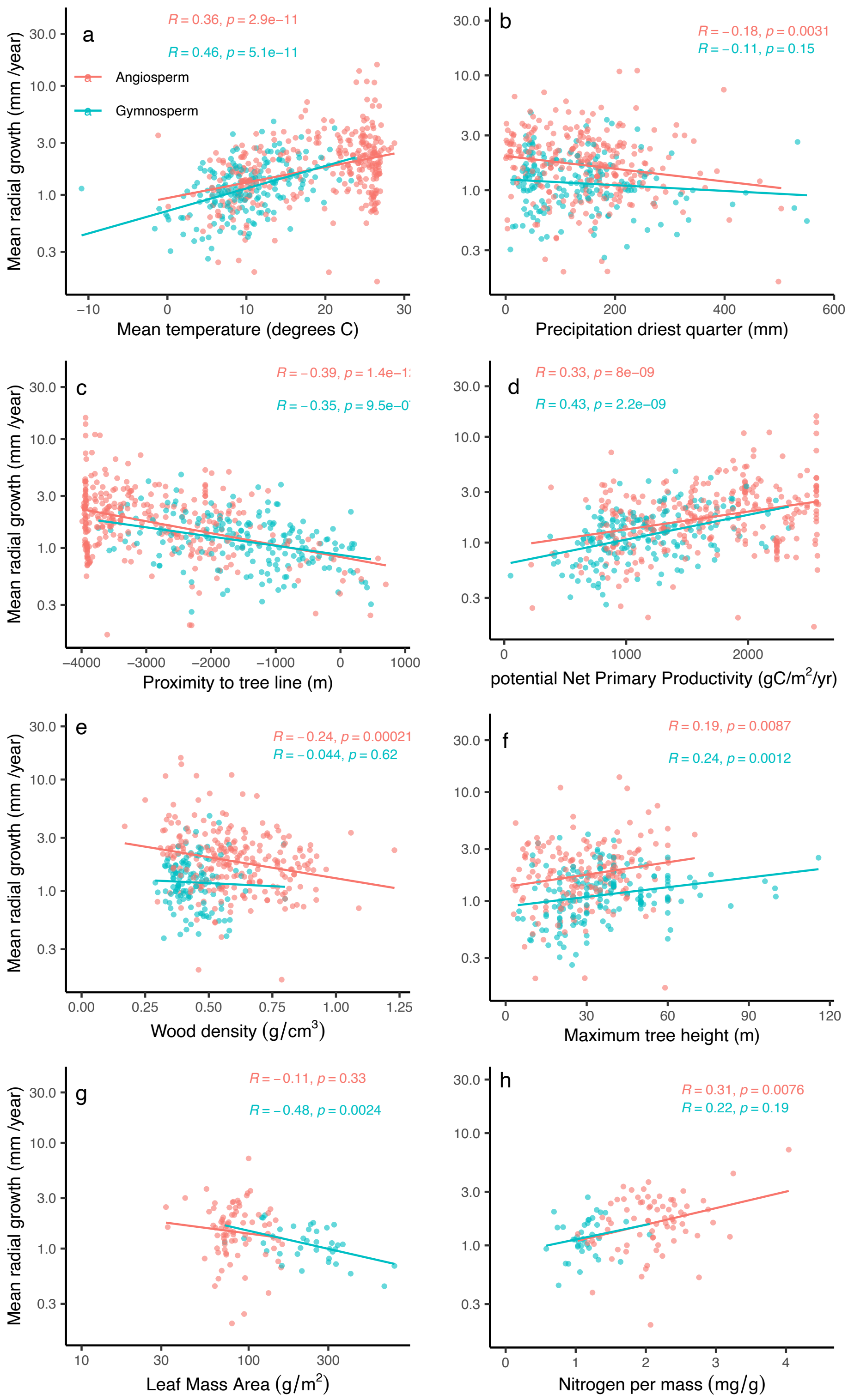

Supplementary Figure 5. Relationships between mean radial growth and most important climate, environmental and trait variables. Mean growth was plotted on a log_{10} scale. Variables include species' mean annual temperature (a) and precipitation during the driest quarter (b), species' mean proximity to the estimated tree line position (c), potential net primary productivity (d), and species' functional traits including wood density (e), maximum species tree height (f), and leaf mass per area (g) and leaf nitrogen (h). Trend lines are fitted using a linear model. Statistics show the correlation coefficient (R) and significance levels (p) for the linear relationships. Data were grouped by the two major taxonomic groups in the dataset, angiosperms and gymnosperms. Note that data for Leaf Mass per Area were log_{10} transformed.

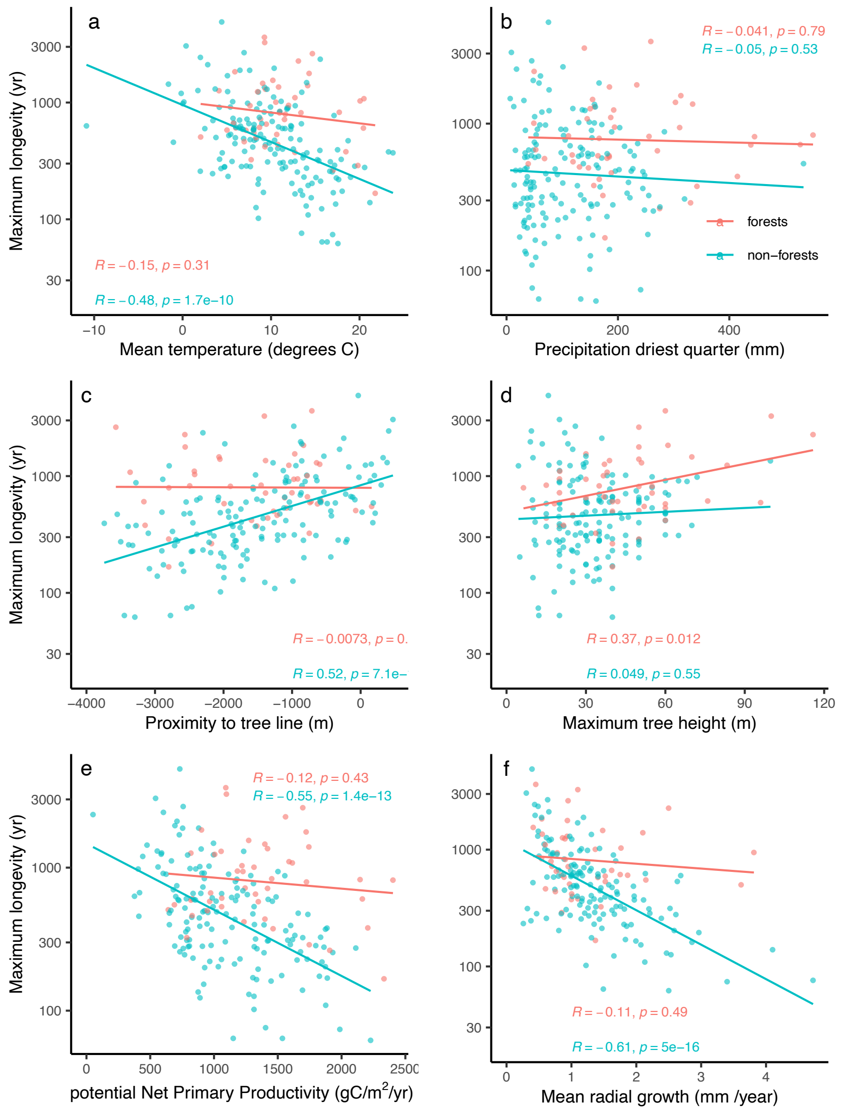

Supplementary Fig. 6 Relationships between tree longevity and mean temperature, precipitation of the driest quarter, proximity to tree line, maximum tree height, site NPP and growth for gymnosperms reaching maximum longevity within forest interiors (“forests”) and those reaching their maximum longevity outside forest interiors (“non-forests). The forest group includes 47 different gymnosperms taxa for which the oldest individuals are found in forest interior environments. This judgment was made by Chris Earle, and species included in this group are: *Abies amabilis, Abies lasiocarpa, Abies magnifica, Abies nordmanniana, Abies pindrow, Abies procera, Abies spectabilis, Agathis australis, Agathis microstachya, Agathis robusta ,Araucaria angustifolia, Araucaria araucana, Araucaria cunninghamii, Araucaria laubenfelsii, Athrotaxis cupressoides, Athrotaxis selaginoides, Callitropsis nootkatensis, Calocedrus decurrens, Chamaecyparis formosensis, Chamaecyparis lawsoniana, Chamaecyparis obtusa, Cryptomeria japonica, Dacrycarpus dacrydioides, Dacrydium cupressinum, Dacrydium elatum, Fitzroya cupressoides, Fokienia hodginsii, Lagarostrobos franklinii, Libocedrus bidwillii, Manoao colensoi, Phyllocladus aspleniifolius, Phyllocladus toatoa, Phyllocladus trichomanoide, Picea glehnii, Picea sitchensis, Picea smithiana, Prumnopitys taxifolia, Sequoia sempervirens, Sequoiadendron giganteum, Taxodium distichum, Taxodium mucronatum, Thuja plicata, Tsuga diversifolia, Tsuga dumosa, Tsuga heterophylla, Tsuga mertensiana, Tsuga sieboldii.*

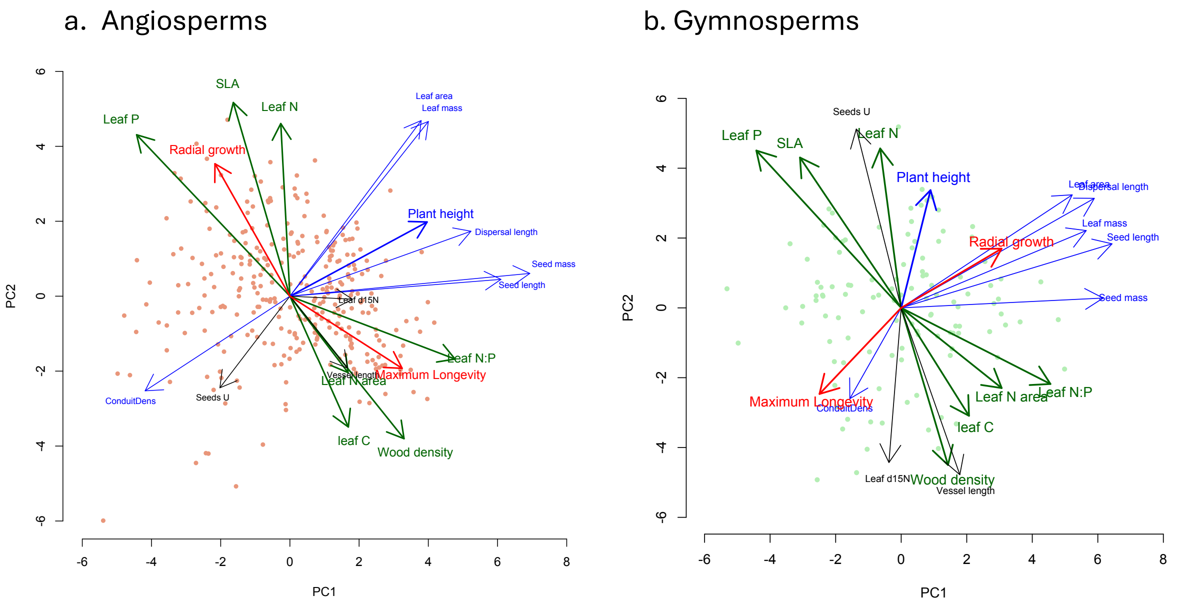

Supplementary Figure 7. Principal component analysis of 17 traits from reference 10. with maximum longevity and ring width from our dataset for angiosperms (panel a, n=297) and gymnosperms (panel b, n=114). All data were log-transformed prior to analysis. Red arrows correspond to maximum species longevity and radial growth from our new dataset, green arrows for traits identified as belonging to plant economics traits (i.e., fast-slow), blue arrows for traits identified as being size related (small-big), and grey for traits for a third group according to reference [12]. Plant economics traits include wood density, Specific Leaf Area (SLA, or 1/LMA), leaf nitrogen content per area (leaf N area), leaf N, P, and C concentrations , leaf N/P ratios (leaf N:P), size related traits include maximum plant height, conduit density, leaf area, leaf fresh mass, seed mass, seed length, dispersal unit length, and the remaining traits are seeds per reproduction unit (seeds U), leaf $\delta^{15}N$, and vessel element length (vessel length).

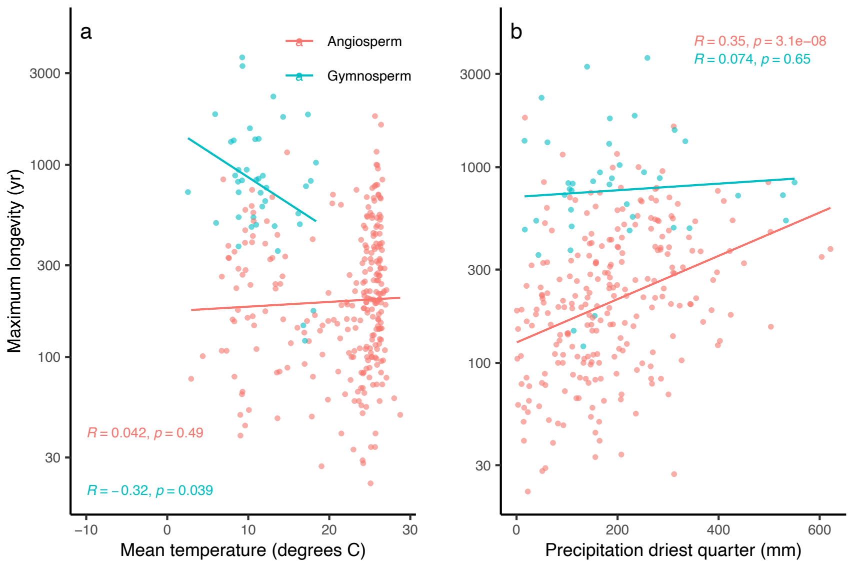

Supplementary Figure 8. Relationships between longevity and the two most important climate, variables at genus level. For each genus we calculated the mean longevity and mean temperature (a) and precipitation during the driest quarter (b). These results show that the key patterns are not driven simply by few widespread genera showing strong relationships with climate but hold up at genus level.

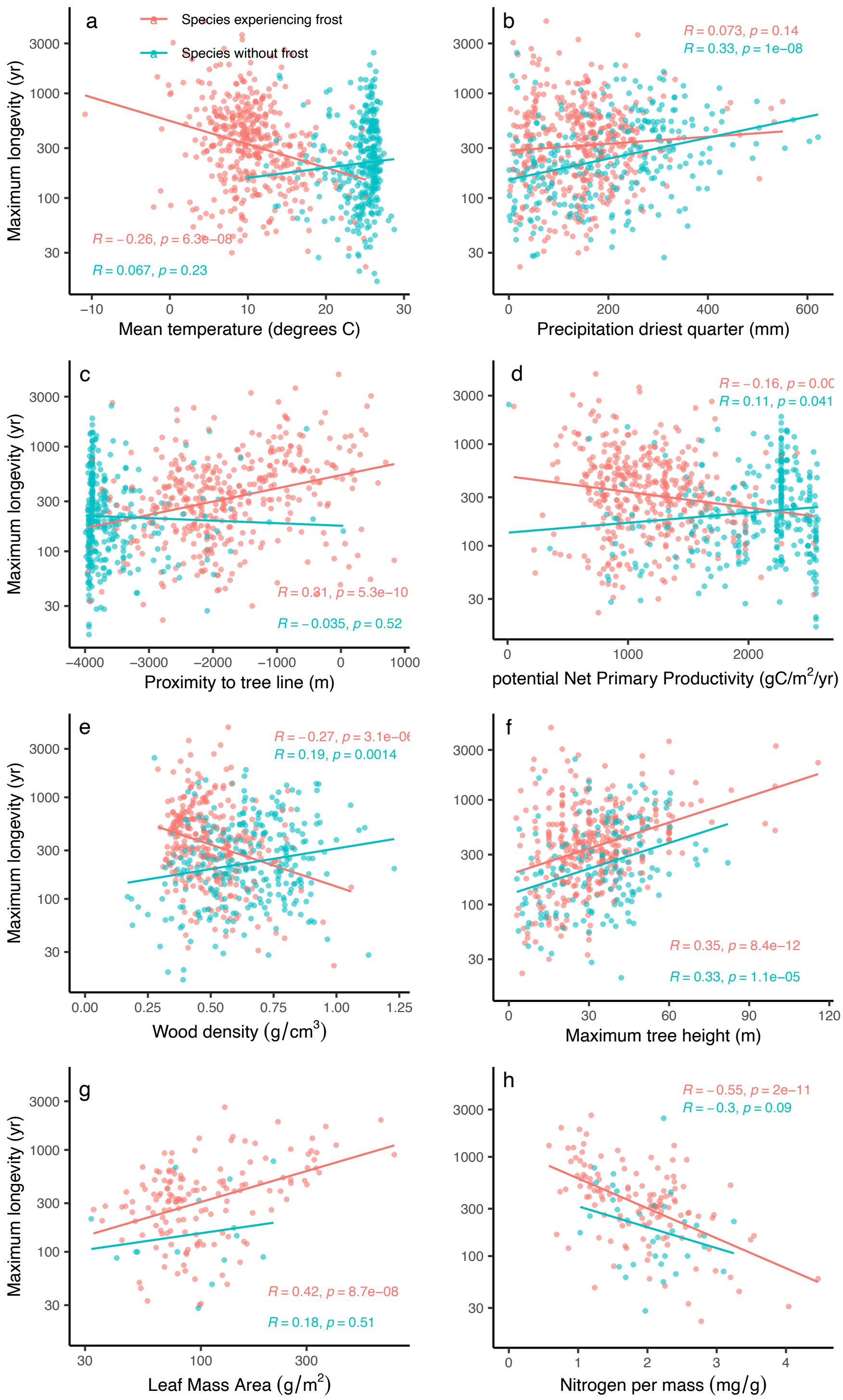

Supplementary Figure 9. Relationships between longevity and all variables shown in main Figure 2 with species separated into those experiencing frost (n= 405) versus species without frost (n= 334). Note that we here combined the entire dataset, irrespective of taxonomic group (angiosperms and gymnosperms). Also note that nearly all gymnosperms (186 out of 205 species) fall into the category of species experiencing frost, and that the category of species with frost mainly consists of angiosperms.

References

1 Grömping, U. Relative importance for linear regression in R: the package relaimpo. *Journal of statistical software* **17**, 1-27 (2007).

2 Jucker, T. *et al.* Tallo: A global tree allometry and crown architecture database. *Global Change Biol.* **28**, 5254-5268 (2022).

3 Zanne, A. E. *et al.* Data from: Towards a worldwide wood economics spectrum. Dryad Digital Repository. doi:10.5061/dryad.234. (2009).

4 Vieilledent, G. *et al.* New formula and conversion factor to compute basic wood density of tree species using a global wood technology database. *Am. J. Bot.* **105**, 1653-1661 (2018).

5 Liu, H. *et al.* Hydraulic traits are coordinated with maximum plant height at the global scale. *Science Advances* **5**, eaav1332 (2019).

6 Sanchez-Martinez, P. *et al.* Increased hydraulic risk in assemblages of woody plant species predicts spatial patterns of drought-induced mortality. *Nature Ecology & Evolution* **7**, 1620-1632 (2023).

7 Zanne, A. E. *et al.* Angiosperm Wood Structure: Global Patterns in Vessel Anatomy and Their Relation to Wood Density and Potential Conductivity. *Am. J. Bot.* **97**, 207-215 (2009). https://doi.org/10.3732/ajb.0900178

8 Wright, I. J. *et al.* The worldwide leaf economics spectrum. *Nature* **428**, 821-827 (2004). https://doi.org/10.1038/nature02403

9 Kattge, J. *et al.* TRY plant trait database - enhanced coverage and open access. *Glob Chang Biol* **26**, 119-188 (2020). https://doi.org/10.1111/gcb.14904

10 Ștefan, V. & Levin, S. plotbiomes: R package for plotting Whittaker biomes with ggplot2 (v1. 0.0). *Zenodo. doi 10.5281/zenodo.7145244* (2018).

11 Mo, L. *et al.* The global distribution and drivers of wood density and their impact on forest carbon stocks. *Nature Ecology & Evolution*, 1-18 (2024).

12 Joswig, J. S. *et al.* Climatic and soil factors explain the two-dimensional spectrum of global plant trait variation. *Nature Ecology & Evolution* **6**, 36-50 (2022).
