# Supplementary material for: Contrasting pathways to tree longevity in gymnosperms and angiosperms
Source: Nat Commun. 2025 Dec 19;17:898. doi: 10.1038/s41467-025-67619-2 (PMC12830884; doi:10.1038/s41467-025-67619-2)
Supplement: Supplementary file 2 — Reporting Summary [file 41467_2025_67619_MOESM2_ESM.pdf]

nature portfolio

Corresponding author(s): Roel Brienen

Last updated by author(s): 2025 11 04

Reporting Summary

Nature Portfolio wishes to improve the reproducibility of the work that we publish. This form provides structure for consistency and transparency in reporting. For further information on Nature Portfolio policies, see our Editorial Policies and the Editorial Policy Checklist.

Statistics

For all statistical analyses, confirm that the following items are present in the figure legend, table legend, main text, or Methods section.

n/a	Confirmed	
☐	☒	The exact sample size (*n*) for each experimental group/condition, given as a discrete number and unit of measurement
☒	☐	A statement on whether measurements were taken from distinct samples or whether the same sample was measured repeatedly
☐	☒	The statistical test(s) used AND whether they are one- or two-sided<br>*Only common tests should be described solely by name; describe more complex techniques in the Methods section.*
☐	☒	A description of all covariates tested
☐	☒	A description of any assumptions or corrections, such as tests of normality and adjustment for multiple comparisons
☐	☒	A full description of the statistical parameters including central tendency (e.g. means) or other basic estimates (e.g. regression coefficient) AND variation (e.g. standard deviation) or associated estimates of uncertainty (e.g. confidence intervals)
☐	☒	For null hypothesis testing, the test statistic (e.g. *F*, *t*, *r*) with confidence intervals, effect sizes, degrees of freedom and *P* value noted<br>*Give P values as exact values whenever suitable.*
☒	☐	For Bayesian analysis, information on the choice of priors and Markov chain Monte Carlo settings
☐	☒	For hierarchical and complex designs, identification of the appropriate level for tests and full reporting of outcomes
☐	☒	Estimates of effect sizes (e.g. Cohen's *d*, Pearson's *r*), indicating how they were calculated

Our web collection on statistics for biologists contains articles on many of the points above.

Software and code

Policy information about availability of computer code

Data collection	No software was used in the collection of data in this study.
Data analysis	All data analysis was performed using R studio (Version 4.4.2). Key R packages used are R packages used in this analysis and their versions are relaimpo, v2.2.7; lavaan, v0.6.19; lavaanPlot, v0.8.1; lme4, v1.1.36; caper, v1.0.3; phytools, v2.5.2; ape, v5.8.1., WorldFlora, 41.14.5, FactorMineR, 2.12, plotbiomes-v1.0.0.

For manuscripts utilizing custom algorithms or software that are central to the research but not yet described in published literature, software must be made available to editors and reviewers. We strongly encourage code deposition in a community repository (e.g. GitHub). See the Nature Portfolio guidelines for submitting code & software for further information.

Data

Policy information about availability of data

All manuscripts must include a data availability statement. This statement should provide the following information, where applicable:

- Accession codes, unique identifiers, or web links for publicly available datasets
- A description of any restrictions on data availability
- For clinical datasets or third party data, please ensure that the statement adheres to our policy

Data on species' maximum longevity, traits, and climate that support the findings of this study are available from https://doi.org/10.6084/m9.figshare.29876984. Source data are provided with this paper. Original raw tree ring data from the ITRDB can be downloaded from https://www.ncei.noaa.gov/products/paleoclimatology/tree-ring, and tropical tree ring data compilations from https://figshare.com/articles/dataset/Locoselli_et_al_2020_Global_tree-

ring_analysis_reveals_rapid_decrease_in_tropical_tree_longevity_with_temperature_PNAS/13119842?file=25178405. Individual longevity records from following oldlists http://www.rmtrr.org/oldlist.htm, https://www.ldeo.columbia.edu/~adk/oldlisteast/, http://www.nativetreesociety.org/dendro/ents_maximum_ages.htm, https://www.oldgrowth.ca/oldtrees/. Tree height data can be downloaded from https://zenodo.org/record/6637599, and maximum height measurements were obtained from https://www.conifers.org and https://Monumentaltrees.com. Wood density data can be obtained from https://zenodo.org/records/13322441, and from https://doi.org/10.18167/DVN1/KRVF0E.

Conduit density from https://doi.org/10.5061/dryad.1138, and conduit density, P50 and HSM from https://datadryad.org/dataset/doi:10.5061/dryad.1138, and from https://www.science.org/doi/10.1126/sciadv.aav1332. Leaf traits from https://www.nature.com/articles/nature02403#Sec15, and seedmass data from https://www.try-db.org/TryWeb/dp.php, database request No 30569. Mean climate and soil data for a species were obtained from the TreeGOER database https://zenodo.org/records/10008994, and gridded climate and elevation data from https://www.worldclim.org/data/worldclim21.html, growing season length and site level Net Primary Productivity (NPP) from https://chelsa-climate.org/.

Species occurrence data from https://doi.org/10.15468/dl.77gcvq.

Research involving human participants, their data, or biological material

Policy information about studies with human participants or human data. See also policy information about sex, gender (identity/presentation), and sexual orientation and race, ethnicity and racism.

Reporting on sex and gender	N/A
Reporting on race, ethnicity, or other socially relevant groupings	N/A
Population characteristics	N/A
Recruitment	N/A
Ethics oversight	N/A

Note that full information on the approval of the study protocol must also be provided in the manuscript.

Field-specific reporting

Please select the one below that is the best fit for your research. If you are not sure, read the appropriate sections before making your selection.

☐ Life sciences ☐ Behavioural & social sciences ☒ Ecological, evolutionary & environmental sciences

For a reference copy of the document with all sections, see nature.com/documents/nr-reporting-summary-flat.pdf

Ecological, evolutionary & environmental sciences study design

All studies must disclose on these points even when the disclosure is negative.

Study description	We compile and analyse global variation in tree longevity for over 700 tree species and assess the covariation between longevity and climate, soil and species traits.
Research sample	Longevity estimates for 739 species obtained from across the globe. Estimates for most species are based on tree ring data from over 530 k trees. Other sources consist of radiocarbon datings, growth projections and historical accounts.
Sampling strategy	Any available sources from either existing databases, or from literature were compiled and thus the strategy was based on data availabilty.
Data collection	Age estimates were obtained from a mixture of tree ring records, radiocarbon dating, growth projections and historical records sourced from original databases and literature. Tree ring data originated from online databases such as the International Tree-Ring databank, and National forest Inventories,which were collected and measured by researchers using standard tree ring methods. These records were further complemented with contributions of original tree ring data from co-authors and collaborators, and with record published in peer reviewed literature, or on well-respected websites such as the OldLists. Studies were excluded if they focussed purely on even-aged plantations, non-natural systems, non-native species, or if they seemed to consist of partially recorded or artificially truncated time series.
Timing and spatial scale	Spatially data cover the entire globe, excluding antarctica and areas without trees. The temporal time span is from ca. 6000 BC to 2020, depending on the time-scale of the available datasets.
Data exclusions	In general, tree ring records with less than 10 series were excluded, but we accepted age estimates based on fewer samples if these can reasonably be believed to represent ages close to species' maxima such as published on Oldlists for commonly sampled species. We only included species listed on the GlobalTreeSearch or those that fit the definition of a tree agreed by IUCN's Global Tree Specialist Groups (GTSG): "a woody plant with usually a single stem growing to a height of at least two meters or if multi-stemmed with then at least one vertical stem five centimetres in diameter at breast height".
Reproducibility	To enesure resproducibility, we provide a detailed description of all the analysis procedures in the Methods section. We furthermore provide the source code and source data or species maximum age on Figshare, allowing for independent reproduction of the findings

of this study.

Randomization

The study is a purely observational study that does not involve experiments, or random treatments and thus randomization is not applicable to this study.

Blinding

Blinding was not relevant to this study given the study uses empircal, quantitative measurments, not subject to personal interpretation. Moreover the collected tree ring data and methods for age estimations are based on standard procedures and can be independently verified.

Did the study involve field work? ☐ Yes ☒ No

Reporting for specific materials, systems and methods

We require information from authors about some types of materials, experimental systems and methods used in many studies. Here, indicate whether each material, system or method listed is relevant to your study. If you are not sure if a list item applies to your research, read the appropriate section before selecting a response.

Materials & experimental systems

n/a	Involved in the study
☒	☐ Antibodies
☒	☐ Eukaryotic cell lines
☒	☐ Palaeontology and archaeology
☒	☐ Animals and other organisms
☒	☐ Clinical data
☒	☐ Dual use research of concern
☒	☐ Plants

Methods

n/a	Involved in the study
☒	☐ ChIP-seq
☒	☐ Flow cytometry
☒	☐ MRI-based neuroimaging

Plants

Seed stocks

No seed stocks were used in this study

Novel plant genotypes

No novel genotypes were used in this study

Authentication

No authebtication of seed stocks, or novel genotypes were involved in this study.
